# Exploring *Isochrysis galbana* Biomass Formats: Effects on Live Prey Oxidative Status and Lipid Profiles for Their Potential Use in Aquaculture Larval Nutrition

**DOI:** 10.1155/anu/8824628

**Published:** 2025-07-27

**Authors:** Ana Galindo, José A. Pérez, Annia Mora, Diana B. Reis, Eduardo Almansa, Ignacio A. Jiménez, Maria Carmo Barreto, Marianna Venuleo, Nieves G. Acosta, Covadonga Rodríguez

**Affiliations:** ^1^Department of Animal Biology, Soil Science and Geology, University of La Laguna, La Laguna, Tenerife, Spain; ^2^Canary Islands Oceanographic Center, Spanish Institute of Oceanography (IEO), CSIC, Santa Cruz de Tenerife, Spain; ^3^Antonio González University Institute of Bio-Organic Chemistry, Department of Organic Chemistry, University of La Laguna, La Laguna, Tenerife, Spain; ^4^Azorean Biodiversity Group and Global Change and Sustainability Institute (CHANGE), Faculty of Sciences and Technology, Centre for Ecology, Evolution and Environmental Changes (cE3c), University of the Azores, Ponta Delgada, Portugal; ^5^Department of Biotechnology, Technological Institute of the Canary Islands, Santa Lucía de Tirajana, Gran Canaria, Spain

**Keywords:** antioxidant capacity, aquaculture industry, *Artemia*, *Isochrysis galbana*, lipid profile, nutritive value, rotifer

## Abstract

Feeding marine larvae commonly relies on live prey, which must be enriched with lipid emulsions and/or microalgae rich in long-chain polyunsaturated fatty acids (LC-PUFA) before being added to larval tanks. This enrichment enhances the nutritional value of live feed and supports larval health and growth. Microalgae are also used in aquaculture as a primary feed source for larvae and for maintaining water quality. However, in-house microalgal cultures are costly, prone to contamination, and require complex harvesting. Sourcing live microalgae from external specialized facilities is also expensive and complicated, as it involves transporting highly diluted biomass, creating a major bottleneck in hatcheries. Consequently, alternative formats of microalgal biomass, including fresh or dried forms, are gaining attention for their ease of use, nutritional stability, and antioxidant potential. To evaluate some of these concentrated biomass substitutes, different formats of *Isochrysis galbana* (spray-dried [ISD], fresh [IFRE], and frozen [IFRO]) were tested on the rotifer *Brachionus plicatilis* and *Artemia*. Prior to the assay, the total antioxidant capacity and lipid composition of the microalgal products were evaluated. Subsequently, live prey were fed the different *I. galbana* formats for 24 h, after which prey survival, lipid composition, and oxidative status were assessed. Our results showed that fresh/frozen *I. galbana* exhibited the highest in vitro antioxidant activity, particularly in the ethyl acetate fraction. However, rotifer survival was compromised when using the IFRE format. The spray-dried microalgae was the best format to enhance phospholipid retention in both zooplankton species, also increasing DHA/EPA (22:6n–3/20:5n–3) ratio and n–3 LC-PUFA content in rotifers. ISD also reduced lipid peroxidation in *Artemia* without negatively affecting the live prey culture. In conclusion, based on lipid composition and antioxidant potential, ISD was the most effective format for feeding rotifers and *Artemia*.

## 1. Introduction

In hatcheries, larval feeding is commonly addressed by using live prey [1], which offer several advantages over formulated diets [2–4]. The rotifer *Brachionus plicatilis* and the crustacean *Artemia* sp. are the primary zooplankton species used to feed fish and crustaceans' larvae [3, 5–7]. However, they are not the natural prey of marine larvae, and their nutritional composition often fails to meet larval physiological needs [6]. Therefore, they are routinely enriched with nutrients, in particular, long chain polyunsaturated fatty acids (LC-PUFA) [8] such as eicosapentaenoic acid (EPA; 20:5n–3), docosahexaenoic acid (DHA; 22:6n–3), and arachidonic acid (ARA; 20:4n–6), which are essential for larval survival and proper development [3]. Nonetheless, achieving consistent levels of key nutrients in enriched live prey remains complex [3]. Rotifers are passive filter feeders, and consequently, their nutritional value can be significantly modified through the diet [9, 10]. In contrast, *Artemia* nauplii metabolize phospholipids into triacylglycerols (TAG) [11] and retroconvert DHA to EPA [12], making DHA enrichment particularly challenging.

Currently, LC-PUFA-rich lipid emulsions are the most commonly used and effective enrichment products [13]. However, their high susceptibility to oxidation can lead to the accumulation of potentially toxic lipid peroxides in larval tissues [8]. Moreover, marine phyto- and zooplankton mainly esterify n–3 LC-PUFA into phospholipids, whereas most commercial lipid emulsions are rich in TAG, which contain lower proportions of n–3 LC-PUFA than polar lipids [14]. Hence, excessive lipid levels are often supplied to live prey, negatively affecting larval digestion and absorption processes [11, 14].

Microalgae are extensively used in both green water applications and live prey enrichment strategies [5, 15]. Microalgal lipid and LC-PUFA contents may be modulated by modifying light intensity, temperature or nutrient availability [16, 17]. In addition, microalgal species offer a wide range of carotenoids and other antioxidant compounds [1], which play an essential role against free radicals generation and lipid oxidation [18]. Nevertheless, in-house microalgae production can be difficult to manage [19], as suboptimal conditions may lead to fluctuating or imbalanced biochemical composition, reduced biomass productivity, and a high risk of culture collapse. Although outsourcing microalgae production eliminates the internal management effort [20], it entails high costs primarily due to transporting large volumes of highly diluted cultures and the risk of receiving contaminated or low-quality batches. This has recently driven the search for viable alternatives such as fresh, frozen or dried microalgae biomass, microencapsulates, cryopreserved or flocculated formats [7]. The costs associated with microalgae downstream processing can be offset by producing more concentrated formats through centrifugation (alone or followed by drying), which are easier to handle and transport, and offer significantly longer shelf life [7]. Fresh biomass can be stored for 2–8 weeks at 4°C [20], while frozen biomass remains viable for up to 2 years at –18°C [21]. On the other hand, dried formats stand out for their ease of use, consistent texture, and extended shelf life of several months [22]. Nevertheless, drying can cause biochemical changes in biomass composition; however, it may also inhibit lipolysis by removing water. As a result, dried biomass tends to maintain a more stable lipid composition over time compared to both fresh and frozen forms [23, 24]. Regardless, fresh and spray-dried formats of various microalgae species have recently been tested for feeding live prey with a certain degree of success [3, 7, 25].

*Isochrysis galbana* has been extensively used to enrich live prey [25, 26]; however, large-scale cultivation requires specific operational and environmental conditions [27]. In this context, the use of concentrated biomass products may offer a practical alternative. This study evaluates novel alternative *I. galbana* biomass formats—spray-dried, fresh, and frozen—as feed for rotifers and *Artemia*, aiming to enhance practical and cost-effective enrichment strategies in larviculture. The assessment focuses on the survival, lipid profile, and oxidative response of the enriched organisms. While spray-dried *I. galbana* has shown promising results in a recent study by our group [25], the potential of fresh and frozen biomass remains unexplored.

## 2. Material and Methods

### 2.1. Microalgal Cultivation and Processing

*I. galbana* BEA 1751B strain (GenBank accession number: MN867789.1), isolated by the Spanish Bank of Algae (BEA; Telde, Gran Canaria, Spain) from natural seawater samples of “Bocacangrejo” saltpans (Agüimes, Gran Canaria, Spain), was transported to the Instituto Tecnológico de Canarias (ITC; Pozo Izquierdo, Gran Canaria, Spain). The microalgae was cultured at the ITC facilities under sterile conditions, starting in 200 mL borosilicate glass flasks and progressively scaled up to 8 L Nalgene polycarbonate carboys, then to 250 and 600 L fiberglass raceways, and finally to 10,000 L PVC-lined raceways. All raceways were located inside a 1500 m^2^ greenhouse. Pure CO_2_ was injected during the daytime (8:00 a.m. to 8:00 p.m.) through a porous ceramic diffuser (0.5–1 L min^−1^) to maintain pH between 7.0 and 8.0. Culture depth was maintained at ≤ 12 cm [25].

*I. galbana* was harvested at the exponential growth phase (biomass concentration ~0.7 g L^−1^) using a continuous-flow disc-stack centrifuge with automatic discharge (Alfalaval, VPNX 510SFD-34G, Alfa Laval Iberia, S.A., Madrid, Spain), yielding the fresh biomass format (IFRE) with a water content of 75%–85% (w/w). A portion of this biomass was frozen at −20°C (frozen format, IFRO), while another portion was dried using a rotary atomizer spray dryer (Ohkawara Kakohki L-12, Ohkawara Kakohki Co., Ltd., Tsuzuki-Ku, Japan) to obtain the spray-dried biomass format (ISD), with a final moisture content of 5%–7% (w/w) [25]. IFRE was tested in its fresh form on live prey, while IFRO was tested after 24 h of storage at −20°. Prior to lipid analyses, IFRE was also frozen; therefore, it is grouped with IFRO in Tables 1 and 2.

### 2.2. Preparation of the Ethanolic Extract and Its Liquid–Liquid Partition

Microalgae formats (25 g) were extracted following Galindo et al. [25]. Briefly, samples were macerated using 96% ethanol (3 × 250 mL × 24 h), filtered and evaporated on a rotary evaporator (Figure S1). Ethanol extracts (7.7 or 3.1 g) were subjected to liquid–liquid partitioning by sequential fractionation using solvents of increasing polarity. The extracts were resuspended in 300 mL distilled water and sequentially extracted with hexane (3 × 300 mL), followed by ethyl acetate (3 × 300 mL). Hexane and ethyl acetate extracts were concentrated using a rotary evaporator, while the aqueous extract was obtained by freeze-drying. Yields from both extraction and partitioning processes are presented in Table S1 (Supporting Information).

### 2.3. Total Antioxidant Activity of *I. galbana* Formats

Extracts, fractions, and Trolox (standard solution) were resuspended in sterile dimethyl sulfoxide at 50 mg mL^−1^ using a sonication bath [25], and then serially diluted in methanol (250–0.244 µg mL^−1^).

Total antioxidant capacity was assessed using the radical scavenging assays 1,1-diphenyl-2-picryl-hydrazyl (DPPH) and 2,2′-azinobis- (3-ethylbenzothiazoline-6-sulfonic acid) (ABTS), with absorbance measured on a BioRad Microplate Reader Model 680 (Bio-Rad Laboratories, Hercules, CA, USA).

DPPH radical scavenging activity was evaluated using a DPPH solution in methanol (45 µg mL^−1^). After reacting with the samples for 30 min in the dark, absorbance was measured at 515 nm [28].

The ABTS solution was prepared by mixing 7 mM ABTS and 2.4 mM potassium persulfate for 12–16 h in the dark at room temperature, followed by dilution with methanol until an absorbance of 0.7 at 734 nm was reached. After 8 min of incubation of ABTS solution with the samples, the absorbance was recorded at 750 nm [29].

The antioxidant activity (%) in both assays was determined by the following equation:

  $$\begin{array}{c} \text{Antioxidant activity}\left(\%\right)=\left[\left({\text{Abs}}_{\text{control}}-{\text{ Abs}}_{\text{sample}}\right)/{\text{Abs}}_{\text{control}}\right]\times 100, \end{array}$$

where Abs_control_ is the ABTS or DPPH radical absorbance in methanol and Abs_sample_ is the absorbance of ABTS or DPPH radical + sample/standard.

IC_50_ values (the concentration required to scavenge 50% of ABTS or DPPH radicals) were determined by extrapolating from the antioxidant activity vs. concentration curve.

### 2.4. Experimental Conditions

The feeding protocols were conducted in triplicate using 5 L cylinder-conical fiberglass tanks over 24 h at the Centro Oceanográfico de Canarias, Instituto Español de Oceanografía (IEO-CSIC, Santa Cruz de Tenerife, Spain) under the rearing conditions described in Table 3. *Artemia* nauplii were obtained from BF *Artemia* cysts (INVE Aquaculture, Dendermonde, Belgium).

Due to the substantially higher moisture content of IFRE and IFRO (75%–85%) compared to ISD (7%–8%), the ISD dose was adjusted to be 10 times higher. Consequently, the feeding treatments tested on rotifers were 0.12 g L^−1^ of ISD and 1.2 g L^−1^ of either IFRE or IFRO. Considering the different live prey densities (Table 3), the *Artemia* feeding assay treatments were added to the culture media at one-tenth the concentration used for rotifers: 0.01 g L^−1^ of ISD, 0.12 g L^−1^ of IFRE and IFRO. Both the microalgae doses and feeding protocols were defined based on previous experience from our research group [11, 25, 30].

### 2.5. Survival and Sample Preparation

Survival was calculated from culture densities (individuals mL^−1^) of rotifer and *Artemia* at the beginning and end of the feeding period. After the feeding experiment, culture media was filtered through 60 µm (rotifers) or 100 µm (*Artemia*) meshes. To remove remaining microalgae, samples were gently washed with seawater. Samples were then rapidly frozen in liquid nitrogen and preserved at −80°C until analysis [25].

### 2.6. Lipid Analysis

The total lipid (TL) contents of samples and products were extracted using chloroform/methanol (2:1, v/v) following the Folch method [31] with modifications [32].

Lipid classes were separated from a TL aliquot using one-dimensional double-development high-performance thin-layer chromatography (HPTLC) [33]. External lipid standards (cod roe lipid extract; digalactosyl-diacylglycerol, monogalactosyl-diacylglycerol, and sulfoquinovosyl-diacylglycerol [Avanti Polar Lipids, Inc., Alabaster, Alabama, USA]) were used to identify the lipid classes [32]. Quantification was carried out by calibrated densitometry [11].

1 mg of TL was submitted to acid-catalyzed transmethylation using toluene and 1% sulfuric acid in methanol (v/v) at 50°C for 16 h in darkness to obtain fatty acid methyl esters (FAMEs) [34]. FAMEs were purified by thin-layer chromatography and subsequently separated and quantified with a TRACE-GC Ultra Gas Chromatograph (Thermo Fisher Scientific Inc., Waltham, Massachusetts, USA) as detailed by Galindo et al. [32]. A mixture of commercial standards (Mix C4-C24 and PUFA No. 3 from menhaden oil [Supelco Inc., Bellefonte, Pennsylvania, USA]), was used to identify FAMEs, and GC-MS (DSQ II, Thermo Fisher Scientific) was employed to confirm their identity when needed.

### 2.7. Antioxidant Response

The peroxides index (PxI) was determined by monitoring ferric chloride (FeCl_3_) synthesis at 500 nm and the use of a standard curve, following Shantha and Decker [35] with slight adjustements [36]. The concentration of lipid peroxides was expressed as meq O_2_ kg^−1^.

Homogenization of samples was developed in ice-cold 20 mM Tris–HCl (w/v) buffer (pH 7.4) in the presence of protease inhibitors (Complete, Merck, Darmstadt, Germany). After centrifugation, supernatants were stored at −80°C until further analysis [36].

Thiobarbituric acid reactive substances (TBARS) assay was conducted to determine the malondialdehyde (MDA) content of rotifer and *Artemia* samples [37]. After the reaction of homogenates with thiobarbituric acid, fluorescence was measured at excitation 530 nm/emission 550 nm (Thermo Scientific Appliskan, Thermo Scientific) [36]. A standard curve of 1,1,3,3-tetramethoxipropane was used to calculate MDA content (nmol MDA mg^−1^ protein).

Antioxidant activity of the enzymes superoxide dismutase (SOD; EC 1.15.1.1), catalase (CAT; EC 1.11.1.6), glutathione reductase (GR; EC 1.6.4.2), and glutathione-S-transferase (GST; EC 2.5.1.18) were calculated in rotifer and *Artemia* fed the microalgae formats.

SOD activity was determined by inhibition of 30 mM pyrogallol auto-oxidation at 420 nm over 10 min, with one unit defined as 50% of inhibition [38]. CAT activity was measured as the decrease in absorbance of 485 mM H_2_O_2_ at 240 nm for 15 min, with a molar extinction coefficient (*ε*) of 42.6 M^−1^ cm^−1^ [39]. GR activity was evaluated with 1 mM GSSG and 60 μM NADPH, monitoring NADPH oxidation at 340 nm for 15 min (*ε* = −6.22 mM^−1^ cm^−1^) [40]. GST activity was measured at 340 nm for 15 min using 5 mM GSH and 1 mM 1-chloro-2,4-dinitrobenzene (CDNB) (*ε* = 9.6 mM^−1^ cm^−1^) [41].

Soluble protein was quantified by using bovine serum albumin as standard [42]. All absorbances were measured in a spectrophotometer and one unit of activity (U) defined as µmol min^−1^.

### 2.8. Statistical Analysis

Data were tested for normality and homoscedasticity, applying arcsine or logarithmic transformations when necessary. Significant differences between treatments were established by one-way ANOVA followed by a Tukey's HSD posthoc test. Welch's test followed by Dunnett's T3 test was developed for no homoscedastic data. In cases of non-normal data distribution, the Kruskall–Wallis nonparametric test was used, followed by pair-wise comparison Mann–Whitney *U* test with Bonferroni correction [43]. Finally, pairwise comparisons were conducted using Student's *t*-test for normally distributed data or the Mann–Whitney test for non-normal data.

Data are presented as means ± standard deviation (SD), with statistical significance set at *p* < 0.05. Statistical analysis were performed using IBM SPSS Statistics 25.0 (IBM Corp., Armonk, NY, USA) for Windows.

## 3. Results

### 3.1. Total Antioxidant Activity of Microalgae Formats

The ethyl acetate extract showed the highest DPPH scavenging capacity across all microalgae formats. Although IFRE/IFRO showed the highest activities in all extracts, only the ethyl acetate fraction was able to inhibit more than 50% of the radicals at 250 µg mL^−1^. Thus, IC_50_ was almost 30-fold less active than Trolox (204.07 ± 4.35 vs. 7.43 ± 0.74 µg mL^−1^, respectively) (Table 4).

Regardless of the microalgae format, the ethyl acetate fractions exhibited the highest ABTS radical scavenging activity. Both these fractions, along with IFRE/IFRO-hexane extract, inhibited more than 50% of ABTS radicals at 250 µg mL^−1^. However, the two organic fractions were 90- to 110-fold less active than Trolox (81.14 ± 1.85, 96.08 ± 2.88, and 96.35 ± 1.73 µg mL^−1^ vs. 0.87 ± 0.18 µg mL^−1^, respectively). IC_50_ of ethyl acetate fraction from IFRE/IFRO was lower than that of the spray-dried (ISD) format (Table 4).

### 3.2. Survival of Live Prey

Rotifer survival was negatively affected by IFRE (39.60% ± 4.41%), while exceeded 85% in rotifers fed ISD and IFRO (100.0% ± 0.0% and 86.8% ± 16.4%, respectively). In contrast, *Artemia* nauplii survival showed no significant differences between dietary treatments (96%–99% in all cases).

### 3.3. Lipid Content and Lipid Classes Profile of Live Prey

The TL content of rotifers remained unchanged across dietary groups (18%–21% DW), with total neutral lipids (TNL) accounting for 81%–83% of TL in rotifers fed ISD and IFRO, and ~87% in those receiving IFRE. The main components of the neutral lipid fraction were monoacylglycerols and diacylglycerols (MAG + DAG) in ISD- and IFRE-rotifers; while TAG were most abundant in IFRO-fed rotifers. FFA were also abundant, with higher levels observed in ISD-fed rotifers compared to those fed IFRO. Both cholesterol (CHO) and sterol esters (SE) were highest in IFRE-rotifers (Table 5).

In contrast, the total polar lipids (TPL) were lowest in IFRE-rotifers (~13% of TL), mainly represented by phosphatidylcholine (PC) in all treatments, with the greatest proportion present in ISD-rotifers. Phosphatidylethanolamine (PE) and phosphatidylglycerol (PG) levels were similar in all treatments. Sphingomyelin (SM) and phosphatidylinositol (PI) were more abundant in ISD-fed rotifers, whereas lysophosphatidylcholine (LPC) was highest in those fed IFRO (Table 5).

*Artemia* lipid content did not significantly differ among dietary treatments (14%–18% DW; Table 6). TNL encompassed between 66% and 68% of TL, with CHO and TAG being the predominant neutral lipid fractions. Notably, MAG + DAG were particularly abundant in *Artemia* fed the IFRE format. In contrast, ISD- and IFRO-fed *Artemia* stand out by their content of FFA, while SE were most abundant in IFRO-*Artemia*.

Regardless of the dietary treatment, PC and PE were the dominant polar lipids classes. PC levels were higher in ISD- and IFRO-fed *Artemia* compared to IFRE, with ISD-fed individuals showing the highest PE content. Finally, LPC content was greater in IFRE-fed *Artemia*, than in the other groups (Table 6).

### 3.4. FA Profile of Live Prey

Table 7 displays the FA composition of rotifers fed with the experimental *I. galbana* diets. Total monounsaturated fatty acids (MUFA) was the primary group of FA in all treatments, followed by total polyunsaturated fatty acids (PUFA) and total saturated fatty acids (SFA). Total n–6 PUFA was highest in ISD-rotifers and lowest in IFRO-rotifers, mainly due to linoleic acid (LA; 18:2n–6). On the other hand, total n–3 PUFA levels were also remarkably high in ISD-rotifers, mainly represented by alpha-linolenic acid (ALA; 18:3n–3) and DHA. By contrast, EPA did not exceed 2.5% in any rotifer group.

The highest DHA/EPA ratio was recorded in ISD (2.68 ± 0.14 vs. 1.6–1.8), while EPA/ARA ratio remained unchanged between 2.1 and 2.3.

Regardless of the dietary treatment, total MUFA was the most abundant group of FA in *Artemia* followed by PUFA and SFA (Table 8). IFRE-*Artemia* exhibited the greatest proportion of total PUFA. Both EPA and DHA levels were similar in all treatments, whereas ALA was highest in IFRE. Total n–6 PUFA and ARA did not significantly vary among dietary treatments whereas LA was more abundant in IFRE-*Artemia* than in the other treatments.

Finally, *Artemia* DHA/EPA (0.02–0.11) and EPA/ARA (3.1–3.6) ratios did not significantly changed with the feeding regime (Table 8).

### 3.5. Live Prey Antioxidant Enzymes and Lipid Peroxidation

**Figure 1:**
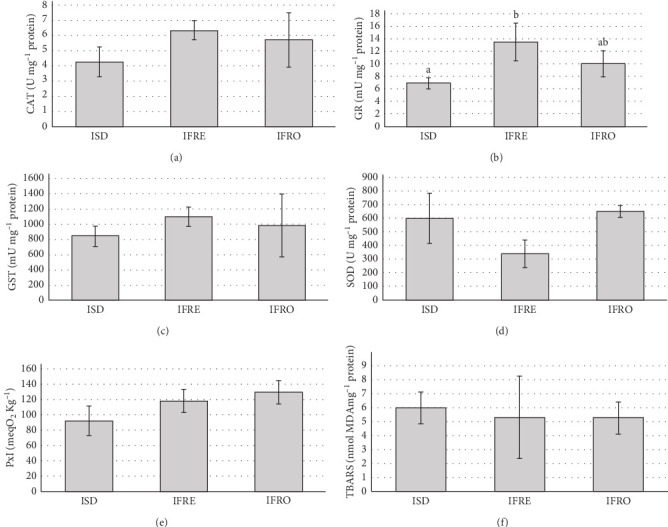
Antioxidant activities: (A) catalase (CAT); (B) glutathione reductase (GR); (C) glutathione-S-transferase (GST); (D) superoxide dismutase (SOD); (E) peroxides index (PxI), and (F) TBARS of rotifers fed the different *Isochrysis galbana* formats. Data are presented as mean ± SD (*n* = 3). IFRE, fresh *I. galbana*; IFRO, frozen *I. galbana*; ISD, spray-dried *I. galbana*. Different letters denote significant differences (*p* < 0.05).

The activities of CAT, GST, and SOD in rotifers did not vary between dietary groups. On the other hand, GR activity ranged from 6.86 ± 0.88 mU mg protein^–1^ in the ISD group to 13.48 ± 3.02 mU mg protein^–1^ in IFRE-rotifers. Moreover, the microalgae format provided to rotifers had no effect on PxI or TBARS levels (Figure 1).

**Figure 2:**
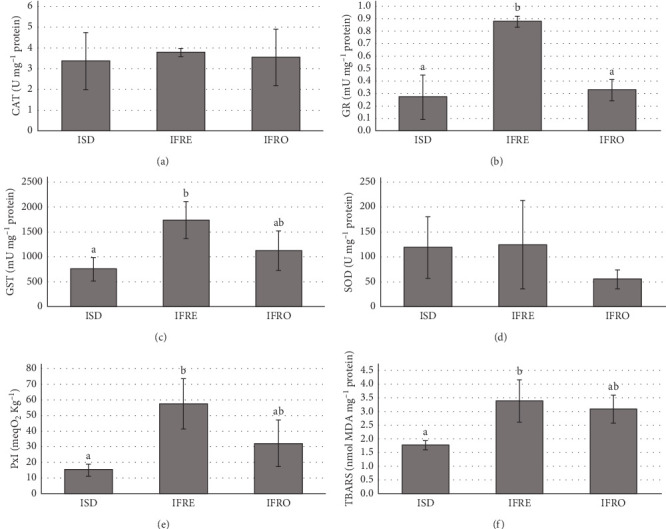
Antioxidant activities: (A) catalase (CAT); (B) glutathione reductase (GR); (C) glutathione-S-transferase (GST); (D) superoxide dismutase (SOD); (E) peroxides index (PxI) and (F) TBARS of *Artemia* fed the different *Isochrysis galbana* formats. Data are presented as mean ± SD (*n* = 3). IFRE, fresh *I. galbana*; IFRO, frozen *I. galbana*; ISD, spray-dried *I. galbana*. Different letters denote significant differences (*p* < 0.05).

On the other hand, GR and GST activities were highest in IFRE-fed *Artemia* (Figure 2). Nonetheless, CAT and SOD activities remained unchanged regardless of the dietary treatment. Importantly, lipid peroxidation indicators, such as PxI and TBARS, were lowest in ISD-fed *Artemia* (15.05 ± 3.85 meq O_2_ Kg^–1^ and 1.76 ± 0.16 nmol MDA mg protein^–1^), followed by those fed the IFRO and IFRE formats.

Table S2 summarizes the main results obtained using the different *I. galbana* formats for feeding rotifers and *Artemia*.

## 4. Discussion

The DPPH radical scavenging activity of *I. galbana* recorded in the present study (25%–61%; Table 4) was higher than previously reported values for this species (28%–35%; using acetone, methanol and hexane) [44], with the fresh/frozen biomass formats showing greater activity than the spray-dried format. Similar results were obtained in our previous assay using fresh/frozen *Navicula salinicola* and the same ISD format [25]. Notably, only the ethyl acetate fraction from the fresh/frozen format was able to scavenge more than 50% of DPPH radicals at 250 µg mL^–1^. Furthermore, the ABTS assay showed IC_50_ below 250 µg mL^–1^ for both ethyl acetate fractions and the hexane fraction from fresh/frozen *I. galbana*. Carotenoids, phenolic compounds and polysaccharides have been described as key compounds contributing to the antioxidant capacity of *I. galbana* [44, 45], with ethyl acetate recommended as the most suitable solvent for extracting FA and carotenoids [46].

*Artemia* survival was not affected by the format of the microalgae biomass used, whereas rotifer survival was seriously compromised by the consumption of IFRE. The doses of IFRE and IFRO were 10-fold higher than that of ISD due to their higher moisture content. This excess of product added to the culture media may have negatively impacted rotifer survival, as some fresh algal biomasses have been described to impair water quality compared to live microalgae [2]. Additionally, the lower survival displayed by rotifers could be also related to reduced nutrient availability, potentially due to the higher ash content in the IFRE format, as suggested by Eryalçın et al. [5].

Phospholipids are highly demanded during the fast growing early stages of marine larvae development, as they are essential structural components of membranes, and play key roles in lipid digestion, absorption and transport [47]. Among them, PC is particularly effective in promoting feeding and growth and it is regarded as the best source of LC-PUFA and DHA for pikeperch larvae and octopus hatchlings, respectively [48, 49]. The ISD format enhanced PC retention in both species, while IFRO did so only in *Artemia*. ISD also increased PI levels in rotifers and PE percentages in *Artemia* (Tables 5 and 6). These effects may be partly due to the greater nutrient availability in spray-dried formats, which have lower ash content [5, 25].

On the other hand, TAG are the primary energy fat source for most marine fish larvae [50]. Adequate ratios between phospholipids and TAG are essential for proper larval development [51]. In this sense, copepods, considered as valuable natural food for marine larvae, have a polar: neutral ratio of ~50:50 [52]. In our experiment, *Artemia* showed a polar: neutral lipid ratio of 32–34/68–66, being more similar to copepods than rotifers (13-19/87-81).

As previously reported in similar studies [53, 54], microalgal dietary treatments did not significantly alter the total lipid content of live prey. However, ISD favored PUFA incorporation into rotifers, despite similar FA profiles across the *I. galbana* formats. Notably, total n-3 LC-PUFA levels were also higher in ISD-rotifers than in those fed IFRE or IFRO, mainly due to DHA (Table 7). A higher retention of total n–3 and DHA in *B. plicatilis* fed spray-dried *Nannochloropsis oculata* compared to freshly microalgae formats has also been reported and attributed to the higher nutrient availability in the spray-dried format [5].

In our experiment, and regardless of the microalgae format, EPA levels in rotifers were lower than those previously reported when fed fresh or frozen *I. galbana* T-ISO (~2% vs. 5%–6%) [55], likely due to the longer enrichment period used in that study (72 vs. 24 h). Nevertheless, our study demonstrated more effectiveness in LC-PUFA incorporation compared to other microalgae feeding strategies. For instance, DHA and EPA were not detected in rotifers and *Artemia* fed with *Chlorella vulgaris* and *Dunaliella salina* for 24 h [56]. Moreover, the proportions of EPA and ARA in *Artemia* were higher than those reported when using the commercial Ori-Green (a microalgae and lipid emulsion-based product) [57]. Differences could be due to the lower LC-PUFA content of *C. vulgaris*, *D. salina* [58] and Ori-Green, although in the case of the latter, its FA profile was not reported in the study.

It has been suggested that microalgae enrichment require a combination with lipid emulsions to successfully increase n–3 LC-PUFA [5]. This was confirmed in our previous study where EPA and DHA levels in rotifers and *Artemia* increased after 5 h of enrichment with ISD combined with a lipid emulsion [25] compared to only ISD for 24 h. Long enrichment periods in *Artemia* could favor the retroconversion of DHA into EPA [13, 59], giving rise to the high EPA values obtained here (Table 8). Shorter enrichment periods combining a DHA-rich microalgae such as *I. galbana* and a lipid emulsion may enhance *Artemia* DHA incorporation and its peroxidation protection [25].

Natural prey such as copepods have DHA/EPA ratios of around 2, which is considered the recommended value for marine larvae nutrition [59]. ISD-rotifers exhibited the highest DHA/EPA ratio (2.68; Table 7), surpassing values reported for rotifers fed either fresh or frozen T-ISO [55], *N. oculata*, or *N. oculata* and *C. vulgaris* [54]. In contrast, *Artemia* exhibited low DHA/EPA ratios (< 0.11) regardless of the microalgae format used, consistent with other studies involving enrichment with lipid emulsions or microalgae and reflecting *Artemia*'s capacity for DHA retroconversion [8, 13, 59].

ARA plays a crucial role in larval growth, survival, pigmentation, and stress tolerance and egg/larval quality. ARA and EPA compete for the same enzymes involved in eicosanoid production. Consequently, the ARA to EPA ratio holds relevant physiological significance in fish nutrition [47, 60, 61], with a recommended value of ~4 for several species [52]. Under our experimental conditions, live prey fed with *I. galbana* exhibited lower ratios, although *Artemia*-ISD tended to approach this value (3.56), without significant differences. Similarly, the enrichment of *Artemia* with microalgae and lipid emulsion showed EPA/ARA ratios of around 4 [25].

LC-PUFA are very susceptible to oxidation, especially under enrichment conditions that involve vigorous aeration, illumination, and elevated temperature [59], which promote the formation of potentially toxic oxidation products [8, 59]. Both rotifers and *Artemia* possess various antioxidant enzymes to metabolize reactive oxygen species (ROS), and to prevent, intercept, and repair damage caused by free radicals, including lipid oxidation [62]. For instance, GST catalyzes the conjugation of GSH with various electrophilic substances, detoxifying endogenous compounds such as peroxidized lipids. Its activity is increased under exogenous chemical sources of oxidative stress as part of the organism´s defensive respponse [63, 64]. In our study, *Artemia*-ISD showed the lowest value of GST activity, suggesting a higher protective effect. On the other hand, GR is involved in the GSH-dependent antioxidative system, catalyzing the reduction of GSSG to GSH [64]. GSH, is a nonenzymatic antioxidant *per se* and a substrate for GST enzyme. The increasing production of ROS can deplete the activity of GSH, inactivating GST, consequently enhancing GR to regenerate GSH and maintain the reduction potential [72]. The IFRE diet in rotifers might be producing an excess of ROS due to the activation of GR, but not of GST (Figure 1). Nevertheless, both GST and GR were enhanced by IFRE treatment in *Artemia* (Figure 2), suggesting that GST was probably upregulated and starting to consume GSH but without being totally depleted.

PxI and TBARS can be also used as indicators of oxidative damage to lipids [35, 59]. IFRE- and IFRO-*Artemia* showed the highest values of PxI and TBARS (Figure 2), paralleling with altered patterns of antioxidant enzymes (higher GR in IFRE and GST in both treatments) as described by Barata et al. [63]. Thus, although fresh/frozen extracts and fractions showed higher activities (Table 4), in vivo results indicate that the ISD format was the most effective in alleviating oxidative stress in *Artemia*, consistent with findings previously reported for rotifers [28]. In this sense, it has been earlier stated that results from in vitro studies assessing the antioxidant capacity of microalgae can be challenging to extrapolate to complex living organisms [65].

Although lipid peroxidation may influence LC-PUFA enrichment efficiency, neither ARA nor DHA appeared to be affected by the higher PxI or TBARS values in *Artemia*, as previously reported by Viciano et al. [59]. In contrast, the high retention of EPA in the ISD group, suggests that this treatment offers greater protection against oxidative stress.

## 5. Conclusion

Both spray-dried and frozen formats seem to be promising for feeding rotifers and *Artemia* without compromising survival. However, the spray-dried format generally resulted in a more favorable DHA/EPA ratio, greater incorporation of n–3 LC-PUFA and phospholipids by live prey, and improved oxidative status compared to the other formats. This enhanced oxidative balance may help preserve LC-PUFA, increasing the nutritional quality and stability of live prey. Consequently, marine larvae may experience improved nutrient assimilation and reduced oxidative stress, fostering healthier development during early life stages. Nonetheless, to optimize enrichment, a lipid emulsion should be used alongside microalgae. In this context, spray-dried *I. galbana* may help mitigate the oxidative stress induced by the lipid emulsion, as already described by our group [25]. Overall, spray-dried *I. galbana* appears more suitable than fresh and frozen formats for live prey enrichment in larval rearing.

## Figures and Tables

**Table 1:** Total lipid content (% dry weight) and lipid class composition (% of total lipid) of the different *I. galbana* formats.

	ISD	IFRE/IFRO
Total lipid	16.2 ± 0.2	15.3 ± 0.2
Lipid classes		
LPC	0.7 ± 0.0	0.7 ± 0.1
PC	2.4 ± 0.8	2.2 ± 0.3
PS + PI	2.2 ± 0.6	1.5 ± 0.3
SQDG	1.3 ± 0.9	0.7 ± 0.3
PE	0.9 ± 0.1	0.9 ± 0.3
DGDG	1.8 ± 0.3	1.3 ± 0.1
MGDG	4.7 ± 1.6	6.1 ± 0.8
TPL^a^	15.7 ± 2.0	14.7 ± 1.2
P	20.6 ± 1.1	25.2 ± 1.0
MAG + DAG	4.3 ± 0.3	4.3 ± 0.2
FFA + PTS	39.5 ± 1.2	34.8 ± 0.5
TAG	11.6 ± 1.5	16.6 ± 0.3
SE	4.3 ± 0.8	4.4 ± 1.2
TNL^a^	63.7 ± 3.1	60.1 ± 2.3

*Note:* Data are presented as means ± SD (*n* = 2).

Abbreviations: DAG, diacylglycerols; DGDG, digalactosyl-diacylglycerol; FFA, free fatty acids; IFRE/IFRO, fresh/frozen *I. galbana*; ISD, spray-dried *I. galbana*; LPC, lysophosphatidylcholine; MAG, monoacylglycerols; MGDG, monogalactosyl-diacylglycerol; P, pigments; PC, phosphatidylcholine; PE, phosphatidylethanolamine; PI, phosphatidylinositol; PS, phosphatidylserine; PTS, phytosterols; SE, sterol esters; SQDG, sulfoquinovosyl-diacylglycerol; TAG, triacylglycerols; TNL, total neutral lipids; TPL, total polar lipids.

^a^Include some minor unidentified lipid classes.

**Table 2:** Total fatty acids (µg mg^–1^ dry weight) and main fatty acid composition (% of total fatty acids) of the different *I. galbana* formats.

	ISD	IFRE/IFRO
Total FA	77.76 ± 0.93	60.41 ± 5.64
Fatty acids		
Total SFA	27.93 ± 1.27	29.35 ± 1.64
14:0	15.48 ± 0.66	17.03 ± 1.01
16:0	11.64 ± 0.42	11.60 ± 0.41
18:0	0.26 ± 0.02	0.18 ± 0.03
Total MUFA	23.66 ± 1.33	24.78 ± 0.10
16:1^a^	7.17 ± 0.41	10.02 ± 0.33
18:1^b^	9.07 ± 0.60	9.50 ± 0.21
20:1^b^	7.29 ± 0.14	5.01 ± 0.54
Total n–6 PUFA	8.79 ± 0.65	9.12 ± 0.84
18:2	3.76 ± 0.03	5.70 ± 0.62
20:4 (ARA)	0.19 ± 0.02	0.19 ± 0.01
22:5	3.91 ± 0.45	2.29 ± 0.17
Total n–3 PUFA	37.04 ± 1.75	33.42 ± 1.19
18:3	6.44 ± 0.01	7.68 ± 0.49
18:4	14.75 ± 0.38	13.15 ± 0.58
20:5 (EPA)	0.47 ± 0.08	0.47 ± 0.11
22:6 (DHA)	15.04 ± 1.16	11.88 ± 0.31
Total PUFA	46.75 ± 2.54	43.66 ± 2.05
EPA/ARA	2.39 ± 0.11	2.53 ± 0.67
DHA/EPA	32.56 ± 3.02	26.10 ± 6.65
Total n–3 LC-PUFA	15.84 ± 1.36	12.60 ± 0.11

*Note:* Data are presented as means ± SD (*n* = 2). Totals include other minor components not shown.

Abbreviations: ARA, arachidonic acid; DHA, docosahexaenoic acid; EPA, eicosapentaenoic acid; FA, fatty acids; IFRE/IFRO, fresh/frozen *I. galbana*; ISD, spray-dried *I. galbana*; LC-PUFA, long chain polyunsaturated fatty acids; MUFA, monounsaturated fatty acids; PUFA, polyunsaturated fatty acids; SFA, saturated fatty acids.

^a^Mainly n–7 isomers.

^b^Mainly n–9 isomers.

**Table 3:** Experimental rearing conditions of rotifer and *Artemia*.

	Rotifer	*Artemia*
Time	24 h	24 h
Tank volume	5 L	5 L
Temperature	22 ± 0.2°C	23 ± 0.3°C
Salinity	28–30 ppt	36 ppt
Density	100 ± 30 individuals mL^–1^	10 ± 1 individuals mL^–1^

**Table 4:** DPPH and ABTS activities (%) and IC_50_ (µg/mL) of the different of *Isochrysis galbana* formats.

	DPPH		ABTS	
	Activity	IC_50_	Activity	IC_50_
ISD				
Ethanol	25.31 ± 0.62^a^	> 250	36.22 ± 1.94^b^	> 250
Hexane	36.76 ± 2.46^a,b^	> 250	37.11 ± 2.11^b^	> 250
Ethyl acetate	38.99 ± 3.99^b^	> 250	74.51 ± 2.35^c^	96.08 ± 2.88
Water	nd	> 250	22.68 ± 4.06^a^	> 250
IFRE/IFRO				
Ethanol	38.55 ± 1.34^a^,*⁣*^*∗*^	> 250	39.11 ± 5.11^a^	> 250
Hexane	48.77 ± 3.32^b^,*⁣*^*∗*^	> 250	58.81 ± 4.12^b^,*⁣*^*∗*^	96.35 ± 1.73
Ethyl acetate	60.98 ± 2.58^c^,*⁣*^*∗*^	204.07 ± 4.35	72.49 ± 3.11^c^	81.14 ± 1.85*⁣*^*∗*^
Water	nd	> 250	42.48 ± 1.09^a,b^,*⁣*^*∗*^	> 250
Trolox	92.21 ± 0.32	7.43 ± 0.74	83.25 ± 1.28	0.87 ± 0.18

*Note:* Data are presented as means ± SD. All determinations were carried out in quadruplicate. Activity (%) was measured at 250 µg mL^−1^ for microalgae extracts and at 100 µg mL^−1^ for Trolox standard.

Abbreviations: ABTS, 2,2'′-azinobis-(3-ethylbenzothiazoline-6-sulfonic acid); DPPH, 1,1-diphenyl-2-picryl-hydrazyl; IC50, concentration yielding 50% scavenging of each radical; IFRE/IFRO, fresh/frozen *I. galbana*; ISD, spray-dried *I. galbana*; nd, not detected.

^a,b,c^ Denote significant differences between solvents within the same format of microalgae (*p*  < 0.05).

*⁣* ^*⁣* ^*∗*^^ Denote significant differences between formats of microalgae for the same solvent (*p*  < 0.05).

**Table 5:** Total lipid (% dry weight) content and lipid class composition (% of total lipid) of rotifers fed with the different *Isochrysis galbana* formats.

	ISD	IFRE	IFRO
Total lipid	20.1 ± 1.6	18.4 ± 0.7	20.8 ± 0.14
Lipid classes			
LPC	0.0 ± 0.0^a^	0.3 ± 0.1^b^	0.9 ± 0.1^c^
SM	0.8 ± 0.1^b^	0.4 ± 0.1^a^	0.3 ± 0.1^a^
PC	6.0 ± 0.6^b^	3.0 ± 0.4^a^	4.3 ± 0.8^a^
PS	0.2 ± 0.1^a^	0.5 ± 0.1^a^	1.2 ± 0.3^b^
PI	2.8 ± 0.6^b^	1.8 ± 0.2^a^	2.4 ± 0.2^a,b^
PG	1.5 ± 0.^3^	1.2 ± 0.4	2.0 ± 0.2
PE	5.4 ± 0.6	4.1 ± 0.6	5.5 ± 0.7
TPL^1^	17.3 ± 1.5^b^	12.6 ± 1.6^a^	18.7 ± 1.6^b^
MAG + DAG	27.0 ± 2.4^b^	25.3 ± 5.1^b^	13.5 ± 1.3^a^
CHO	9.5 ± 1.6^a^	12.8 ± 0.8^b^	10.9 ± 0.8^a,b^
FFA	24.2 ± 2.4^b^	20.4 ± 1.2^a,b^	17.7 ± 2.7^a^
TAG	17.2 ± 2.5^a^	17.2 ± 2.0^a^	32.2 ± 1.0^b^
SE	4.7 ± 0.7^a^	11.6 ± 1.8^b^	5.8 ± 3.6^a,b^
TNL^1^	82.7 ± 1.5^a^	87.4 ± 1.6^b^	81.3 ± 1.6^a^

*Note:* Data are presented as means ± SD (*n* = 3). Different letters in superscript within the same row denote significant differences between dietary treatments (*p*  < 0.05).

Abbreviations: CHO, cholesterol; DAG, diacylglycerols; FFA, free fatty acids; IFRE, fresh *I. galbana*; IFRO, frozen *I. galbana*; ISD, spray-dried *I. galbana*; LPC, lysophosphatidylcholine; MAG, monoacylglycerols; PC, phosphatidylcholine; PE, phosphatidylethanolamine; PG, phosphatidylglycerol; PI, phosphatidylinositol; PS, phosphatidylserine; SE, sterol esters; SM, sphingomyelin; TAG, triacylglycerols; TNL, total neutral lipids; TPL, total polar lipids.

^1^Include some minor unidentified lipid classes.

**Table 6:** Total lipid content (% dry weight) and lipid class composition (% of total lipid) of *Artemia* fed with the different *Isochrysis galbana* formats.

	ISD	IFRE	IFRO
Total lipid	17.6 ± 1.6	16.0 ± 2.2	14.44 ± 3.1
Lipid classes			
LPC	0.2 ± 0.2^a^	0.7 ± 0.1^b^	0.3 ± 0.1^a^
SM	0.6 ± 0.3	0.8 ± 0.2	0.8 ± 0.4
PC	11.8 ± 0.6^b^	10.1 ± 0.7^a^	13.2 ± 0.3^b^
PS	2.1 ± 0.3	1.6 ± 0.3	2.2 ± 0.0
PI	4.4 ± 0.4	3.5 ± 0.5	3.5 ± 0.0
PG	2.8 ± 0.3	2.8 ± 0.8	2.7 ± 0.0
PE	11.1 ± 0.9^b^	8.7 ± 0.9^a^	9.2 ± 0.2^a^
TPL^1^	34.2 ± 2.2	32.5 ± 1.6	34.4 ± 1.2
MAG + DAG	8.8 ± 1.7^a^	18.0 ± 4.0^b^	4.4 ± 0.7^a^
CHO	23.1 ± 4.0	19.9 ± 0.4	20.0 ± 1.8
FFA	10.5 ± 2.1^b^	4.0 ± 0.9^a^	7.0 ± 0.4^b^
TAG	17.9 ± 0.5	22.2 ± 1.9	21.2 ± 3.1
SE	5.5 ± 1.0^a^	3.4 ± 0.4^a^	12.0 ± 1.1^b^
TNL^1^	65.8 ± 2.2	67.5 ± 1.6	65.6 ± 1.2

*Note:* Data are presented as means ± SD (*n* = 3). Different letters in superscript within the same row denote significant differences between dietary treatments (*p* < 0.05).

Abbreviations: CHO, cholesterol; DAG, diacylglycerols; FFA, free fatty acids; IFRE, fresh *I. galbana*; IFRO, frozen *I. galbana*; ISD, spray-dried *I. galbana*; LPC, lysophosphatidylcholine; MAG, monoacylglycerols; PC, phosphatidylcholine; PE, phosphatidylethanolamine; PG, phosphatidylglycerol; PI, phosphatidylinositol; PS, phosphatidylserine; SE, sterol esters; SM, sphingomyelin; TAG, triacylglycerols; TNL, total neutral lipids; TPL, total polar lipids.

^1^Include some minor unidentified lipid classes.

**Table 7:** Total fatty acids (µg mg^−1^ dry weight) and main fatty acid composition (% of total fatty acids) of rotifers fed the different *I. galbana* formats.

	ISD	IFRE	IFRO
Total FA	46.43 ± 11.81	54.69 ± 5.88	67.56 ± 7.06
Fatty acids			
Total SFA	26.79 ± 3.72	22.91 ± 1.68	23.91 ± 1.96
14:0	4.51 ± 0.63	4.76 ± 0.33	5.06 ± 0.19
16:0	11.88 ± 1.26	9.92 ± 0.59	10.30 ± 0.68
18:0	9.11 ± 1.98	7.05 ± 1.48	7.11 ± 2.39
Total MUFA	36.90 ± 2.45	44.43 ± 5.71	43.88 ± 1.70
16:1^1^	13.71 ± 1.00	15.08 ± 2.60	14.42 ± 0.32
18:1^2^	17.83 ± 1.21^a^	23.28 ± 2.26^b^	22.68 ± 0.95^b^
20:1^2^	3.45 ± 0.47	4.04 ± 0.73	4.50 ± 0.68
Total n−6 PUFA	8.09 ± 0.65^b^	6.27 ± 0.79^a,b^	5.17 ± 0.41^a^
18:2	3.08 ± 0.06^b^	3.50 ± 0.52^b^	2.17 ± 0.23^a^
20:4 (ARA)	1.19 ± 0.25	1.18 ± 0.07	0.96 ± 0.06
Total n−3 PUFA	15.82 ± 1.22^b^	11.95 ± 0.89^a^	13.16 ± 0.88^a^
18:3	2.66 ± 0.32^b^	1.89 ± 0.30^a^	1.88 ± 0.13^a^
20:5 (EPA)	2.47 ± 0.05	2.47 ± 0.52	2.15 ± 0.18
22:6 (DHA)	6.62 ± 0.87^b^	3.89 ± 0.47^a^	3.75 ± 0.26^a^
Total PUFA	28.41 ± 2.02^b^	23.58 ± 0.16^a^	23.58 ± 1.39^a^
EPA/ARA	2.15 ± 0.45	2.08 ± 0.31	2.26 ± 0.27
DHA/EPA	2.68 ± 0.14^b^	1.59 ± 0.14^a^	1.75 ± 0.14^a^
Total n−3 LC-PUFA	9.10 ± 0.82^b^	6.36 ± 0.99^a^	5.90 ± 0.38^a^

*Note:* Data are presented as means ± SD (*n* = 3). Different letters in superscript within the same row denote significant differences between dietary treatments (*p* < 0.05). Totals include other minor components not shown.

Abbreviations: ARA, arachidonic acid; DHA, docosahexaenoic acid; EPA, eicosapentaenoic acid; FA, fatty acids; IFRE, fresh *I. galbana*; IFRO, frozen *I. galbana*; ISD, spray-dried *I. galbana*; LC-PUFA, long chain polyunsaturated fatty acids; MUFA, monounsaturated fatty acids; PUFA, polyunsaturated fatty acids; SFA, saturated fatty acids.

^1^Mainly n−7 isomers.

^2^Mainly n−9 isomers.

**Table 8:** Total fatty acids (µg mg^−1^ dry weight) and main fatty acid composition (% of total fatty acids) of *Artemia* fed the different *I. galbana* formats.

	ISD	IFRE	IFRO
Total FA	90.28 ± 15.74	64.65 ± 8.92	64.57 ± 26.02
Fatty acids			
Total SFA	22.84 ± 2.00^b^	19.57 ± 0.66^a^	20.25 ± 0.51^a,b^
14:0	0.96 ± 0.14^a,b^	1.20 ± 0.11^b^	0.88 ± 0.05^a^
16:0	10.64 ± 0.66	9.67 ± 0.33	9.87 ± 0.26
18:0	8.99 ± 1.35^b^	6.42 ± 0.28^a^	7.37 ± 0.23^a,b^
Total MUFA	49.31 ± 3.04^a,b^	49.35 ± 1.15^a^	53.40 ± 0.96^b^
16:1^1^	10.66 ± 2.17	11.52 ± 1.12	12.40 ± 0.53
18:1^2^	36.80 ± 0.70^a^	36.36 ± 0.87^a^	39.43 ± 0.59^b^
20:1^2^	0.81 ± 0.20	0.75 ± 0.07	0.81 ± 0.20
Total n−6 PUFA	8.28 ± 0.24	8.88 ± 0.35	8.03 ± 0.50
18:2	4.89 ± 0.09^a^	5.72 ± 0.23^b^	4.94 ± 0.13^a^
20:4 (ARA)	3.05 ± 0.16	2.84 ± 0.14	3.09 ± 0.39
Total n−3 PUFA	14.41 ± 0.50	16.24 ± 1.65	14.23 ± 0.32
18:3	2.86 ± 0.09^a^	4.01 ± 0.18^c^	3.22 ± 0.12^b^
20:5 (EPA)	10.82 ± 0.32^b^	8.97 ± 0.31^a^	9.39 ± 0.31^a^
22:6 (DHA)	0.20 ± 0.10	1.00 ± 0.65	0.45 ± 0.28
Total PUFA	25.00 ± 0.38^a^	27.22 ± 1.41^b^	24.37 ± 0.48^a,b^
EPA/ARA	3.56 ± 0.26	3.17 ± 0.24	3.07 ± 0.38
DHA/EPA	0.02 ± 0.01	0.11 ± 0.07	0.05 ± 0.03
Total n−3 LC-PUFA	11.02 ± 0.42	10.26 ± 1.12	9.85 ± 0.06

*Note:* Data are presented as means ± SD (*n* = 3). Different letters in superscript within the same row denote significant differences between dietary treatments (*p* < 0.05). Totals include other minor components not shown.

Abbreviations: ARA, arachidonic acid; DHA, docosahexaenoic acid; EPA, eicosapentaenoic acid; FA, fatty acids; IFRE, fresh *I. galbana*; IFRO, frozen *I. galbana*; ISD, spray-dried *I. galbana*; LC-PUFA, long chain polyunsaturated fatty acids; MUFA, monounsaturated fatty acids; PUFA, polyunsaturated fatty acids; SFA, saturated fatty acids.

^1^Mainly n−7 isomers.

^2^Mainly n−9 isomers.

## Data Availability

The data that support the findings of this study are available in the article and Suppporting Information.

## Supporting Information

Supporting Information Figure S1. Flowchart of ethanolic extraction and liquid–liquid partition fractions from *Isochrysis galbana* [25]. Table S1. Yield of crude extracts and fractions obtained from Isochrysis galbana via maceration and liquid–liquid partition. Table S2. Summary of key experimental results on survival, lipid classes, fatty acid composition, antioxidant activities, and lipid peroxidation indicators in rotifers and *Artemia* fed the different Isochrysis galbana formats.
