# Supplementary material for: Exploring Isochrysis galbana Biomass Formats: Effects on Live Prey Oxidative Status and Lipid Profiles for Their Potential Use in Aquaculture Larval Nutrition
Source: Aquac Nutr. 2025 Jul 27;2025:8824628. doi: 10.1155/anu/8824628 (PMC12318626; doi:10.1155/anu/8824628)
Supplement: Supporting Information — Figure S1. Flowchart of ethanolic extraction and liquid–liquid partition fractions from Isochrysis galbana [25]. Table S1. Yield of crude extracts and fractions obtained from Isochrysis galbana via maceration and liquid–liquid partition. Table S2. Summary of key experimental results on survival, lipid classes, fatty acid composition, antioxidant activities, and lipid peroxidation indicators in rotifers and Artemia fed the different Isochrysis galbana formats. [file 8824628.f1.docx]

Exploring *Isochrysis galbana* biomass formats: Effects on live prey oxidative status and lipid profiles for their potential use in aquaculture larval nutrition.

Ana Galindo ^1,2^, José A. Pérez ^1,*^, Annia Mora ^1^, Diana B. Reis ^1^, Eduardo Almansa^2^, Ignacio A. Jiménez ^3^, Maria Carmo Barreto ^4^, Marianna Venuleo ^5^, Nieves G. Acosta ^1^ and Covadonga Rodríguez ^1^.

^1^ Departamento de Biología Animal, Edafología y Geología, Universidad de La Laguna. Avenida Astrofísico Francisco Sánchez s/n, 38206 La Laguna, Tenerife, Spain. agalindg@ull.edu.es; janperez@ull.edu.es; anniamoramartin@gmail.com; dbotelho@ull.edu.es; [ngacosta@ull.edu.es](mailto:ngacosta@ull.edu.es); covarodr@ull.edu.es

^2^ Centro Oceanográfico de Canarias, Instituto Español de Oceanografia (IEO), CSIC. Calle Farola del Mar, 22, Dársena pesquera de San Andrés, 38180 Santa Cruz de Tenerife, Spain. eduardo.almansa@ieo.csic.es

^3^ Instituto Universitario de Bio-Orgánica Antonio González, Departamento de Química Orgánica, Universidad de La Laguna. Avenida Astrofísico Francisco Sánchez 2, 38206 La Laguna, Tenerife, Spain. ignadiaz@ull.edu.es

^4^ University of the Azores, Faculty of Sciences and Technology, Centre for Ecology, Evolution and Environmental Changes (cE3c), Azorean Biodiversity Group & Global Change and Sustainability Institute (CHANGE), 9501-321 Ponta Delgada, Portugal. maria.cr.barreto@uac.pt

^5^ Departamento de Biotecnología, División de Investigación y Desarrollo Tecnológico, Instituto Tecnológico de Canarias. Playa de Pozo Izquierdo, s/n, 35119 Santa Lucía de Tirajana, Gran Canaria, Spain. [mvenuleo@itccanarias.org](mailto:mvenuleo@itccanarias.org)

***** Correspondence: janperez@ull.edu.es; Tel.: +34 922318340

**
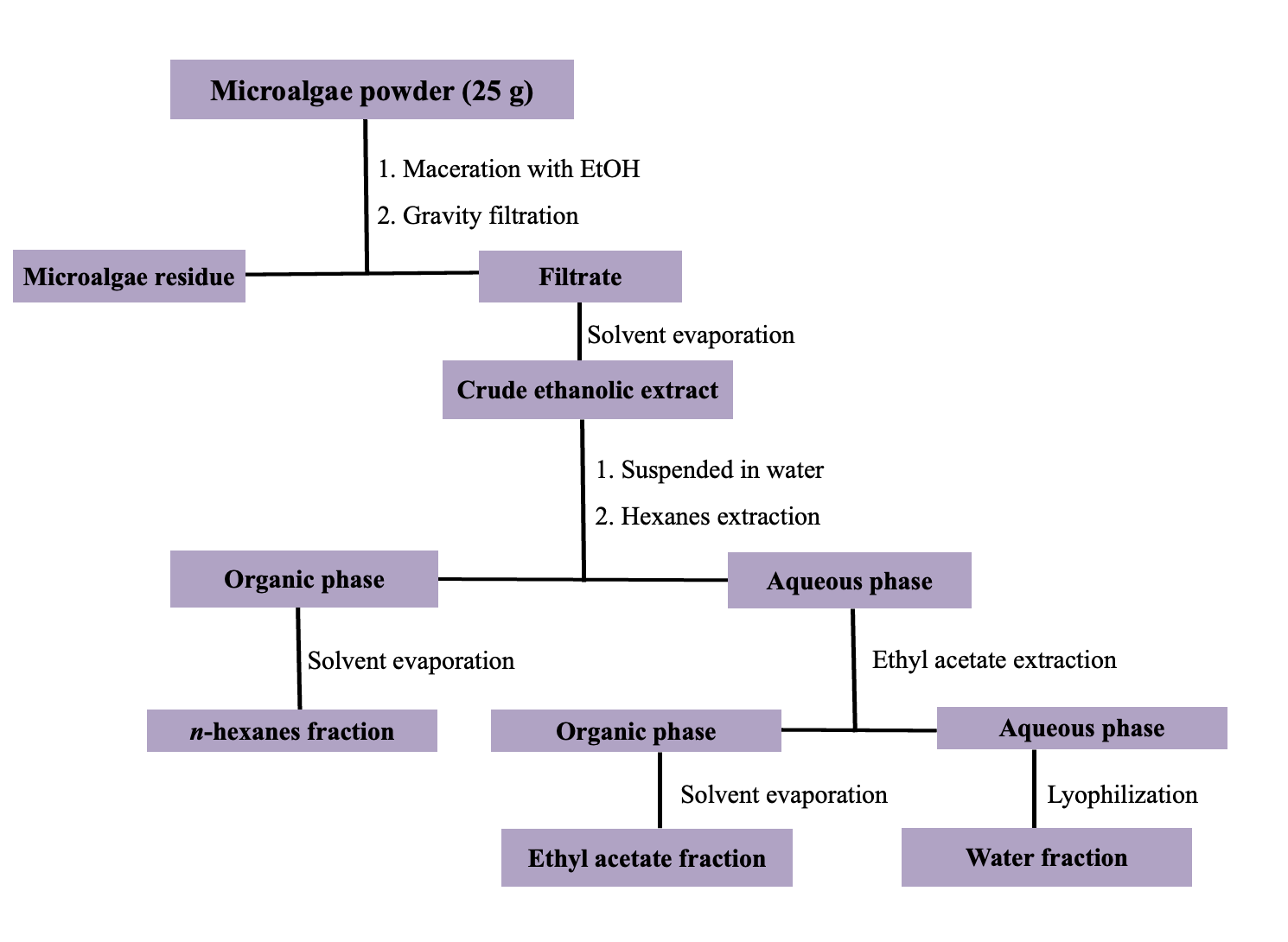
Figure S1.**

**Fig. S1.** Flowchart of ethanolic extraction and liquid-liquid partition fractions from *Isochrysis galbana* (Galindo et al., 2024).

**Table S1.** Yield of crude extracts and fractions obtained from *Isochrysis galbana* via maceration and liquid-liquid partition^1^.

| **Microalgae format** | **Crude/fractions** | **Weight (g)** | **Yield (% DW)^2^** |
| --- | --- | --- | --- |
| ISD | Ethanol | 7.70 | 30.80 |
|  | Hexane | 2.49 | 12.01 |
|  | Ethyl acetate | 0.83 | 4.01 |
|  | Water | 5.96 | 28.74 |
| IFRE/IFRO*^3^* | Ethanol | 3.10 | 25.00 |
|  | Hexane | 1.21 | 9.74 |
|  | Ethyl acetate | 0.25 | 2.04 |
|  | Water | 2.53 | 20.42 |

^1^Macerations were performed using 25 g of microalgae. ^2^Percentage yield (%) = [amount (g) of the dry extract or fraction/amount of the dry sample (25 g)] x 100. ^3^Fresh *I. galbana* was frozen prior to the analysis of the antioxidant activity, and thus it is presented together with frozen *I. galbana*.

|  | **Rotifer** | | | | | |  | ***Artemia*** | | | | | | |
| --- | --- | --- | --- | --- | --- | --- | --- | --- | --- | --- | --- | --- | --- | --- |
|  | **ISD** |  | **IFRE** |  | **IFRO** |  |  | **ISD** |  | **IFRE** |  | **IFRO** |  |  |
| *Survival (%)* | 100.0 ± 0.0 | ^b^ | 39.6 ± 4.4 | ^a^ | 86.8 ± 16.4 | ^b^ |  | 97.5 ± 4.3 |  | 99.2 ± 1.3 |  | 96.3 ± 6.3 |  |  |
|  |  |  |  |  |  |  |  |  |  |  |  |  |  |  |
| *Lipid classes (%)* |  |  |  |  |  |  |  |  |  |  |  |  |  |  |
| PC | 6.0 ± 0.6 |  | 3.0 ± 0.4 |  | 4.3 ± 0.8 |  |  | 11.8 ± 0.6 | ^b^ | 10.1 ± 0.7 | ^a^ | 13.2 ± 0.3 | ^b^ |  |
| PE | 5.4 ± 0.6 |  | 4.1 ± 0.6 |  | 5.5 ± 0.7 |  |  | 11.1 ± 0.9 | ^b^ | 8.7 ± 0.9 | ^a^ | 9.2 ± 0.2 | ^a^ |  |
| *Fatty acids* *(%)* |  |  |  |  |  |  |  |  |  |  |  |  |  |  |
| EPA | 2.5 ± 0.0 |  | 2.5 ± 0.5 |  | 2.1 ± 0.2 |  |  | 10.8 ± 0.3 | ^b^ | 9.0 ± 0.3 | ^a^ | 9.4 ± 0.3 | ^a^ |  |
| DHA | 6.6 ± 0.9 | ^b^ | 3.9 ± 0.5 | ^a^ | 3.7 ± 0.3 | ^a^ |  | 0.2 ± 0.1 |  | 1.0 ± 0.6 |  | 0.4 ± 0.3 |  |  |
| DHA/EPA | 2.7 ± 0.14 | ^b^ | 1.6 ± 0.1 | ^a^ | 1.7 ± 0.1 | ^a^ |  | 0.02 ± 0.01 |  | 0.11 ± 0.07 |  | 0.05 ± 0.03 |  |  |
| Total PUFA | 28.4 ± 2.0 | ^b^ | 23.6 ± 0.2 | ^a^ | 23.6 ± 1.4 | ^a^ |  | 25.0 ± 0.4 | ^a^ | 27.2 ± 1.4 | ^b^ | 24.4 ± 0.5 | ^ab^ |  |
| Total n-3 LC-PUFA | 9.1 ± 0.8 | ^b^ | 6.4 ± 1.0 | ^a^ | 5.9 ± 0.3 | ^a^ |  | 11.0 ± 0.4 |  | 10.3 ± 1.1 |  | 9.8 ± 0.1 |  |  |
| *Antioxidant activities (mU mg^-1^ protein)* | |  |  |  |  |  |  |  |  |  |  |  |  |  |
| GR | 6.9 ± 0.9 | ^a^ | 13.5 ± 3.0 | ^b^ | 10.0 ± 2.1 | ^ab^ |  | 0.3 ± 0.2 | ^a^ | 0.9 ± 0.0 | ^b^ | 0.3 ± 0.1 | ^a^ |  |
| GST | 844 ± 127 |  | 1101 ± 412 |  | 985 ± 320 |  |  | 753 ± 234 | ^a^ | 1736 ± 368 | ^b^ | 1127 ± 396 | ^ab^ |  |
| *Lipid peroxidation* |  |  |  |  |  |  |  |  |  |  |  |  |  |  |
| PxI (meq O_2_ kg^-1^) | 92.2 ± 19.1 |  | 118.1 ± 15.2 |  | 129.5 ± 15.2 |  |  | 15.0 ± 3.8 | ^a^ | 57.5 ± 16.1 | ^b^ | 32.1 ± 15.0 | ^ab^ |  |
| TBARS (nmol MDA mg^-1^ protein) | 6.0 ± 1.1 |  | 5.3 ± 2.9 |  | 5.3 ± 1.1 |  |  | 1.8 ± 0.2 | ^a^ | 3.4 ± 0.8 | ^b^ | 3.1 ± 0.5 | ^b^ |  |

**Table S2.** Summary of key experimental results on survival, lipid classes and fatty acid composition, antioxidant activities and lipid peroxidation indicators in rotifers and *Artemia* fed the different *Isochrysis galbana* formats.

Data are presented as mean ± SD (n=3). Different letters in superscript within the same row denote significant differences between dietary treatments (p<0.05) for each species. ISD, spray-dried *I. galbana*; IFRE, fresh *I. galbana*; IFRO, frozen *I. galbana*.; PC, phosphatidylcholine; PE, phosphatidylethanolamine; EPA, eicosapentaenoic acid; DHA, docosahexaenoic acid; PUFA, polyunsaturated fatty acids; LC-PUFA, long chain polyunsaturated fatty acids; GR, glutathione reductase; GST, glutathione-S-transferase; PxI, peroxides index.
